# Evidence Synthesis of Mobile-Based Smoking Cessation Interventions for Adult Non-Pregnant Smokers and Practice Recommendations Based on the Chinese Context

**DOI:** 10.3390/healthcare14213644

**Published:** 2026-10-27

**Authors:** Xia Luo, Xingwang Qiu, Yuancen Shi, Hua Guan

**Affiliations:** 1 School of Medicine, University of Electronic Science and Technology of China, Chengdu 610054, China; luoxia@med.uestc.edu.cn (X.L.);; 2 Sichuan Academy of Medical Sciences & Sichuan Provincial People’s Hospital (Affiliated Hospital of University of Electronic Science and Technology of China), Chengdu 610072, China

**Keywords:** mHealth, smoking cessation, smokers, evidence-based practice, evidence summary, Chinese practice

## Abstract

**Introduction**: Tobacco dependence is a leading preventable cause of death globally and in China. Traditional cessation services face low coverage, poor accessibility, and suboptimal adherence. Mobile health interventions—SMS and smartphone apps—offer scalable, personalized alternatives and have proven effective. However, translating evidence to China faces two bottlenecks: locally available apps lack efficacy validation in Chinese populations, and integration pathways with existing services remain undefined. This study synthesized best evidence on mobile-based smoking cessation interventions for adult non-pregnant smokers and formulated contextualized recommendations for China. **Methods**: Following evidence-based medicine methodologies, we systematically searched clinical decision support systems, Chinese and English databases, guideline websites, and professional association websites for literature published up to 31 August 2026. Literature quality was assessed using rigorous tools, and evidence was graded according to the Joanna Briggs Institute system. **Results**: 22 publications were included, comprising 2 clinical decision, 5 guidelines, 1 expert consensus, 12 systematic reviews, 1 statistical report, and 1 randomized controlled trial. A total of 28 best evidence items were synthesized. **Conclusions**: This study summarizes best evidence on role orientation, mechanisms, functionalities, and intervention strategies of mobile-based smoking cessation for adult non-pregnant smokers. The findings, combined with China-specific recommendations, provide valuable references for local development. **Implications**: Mobile cessation interventions should be integrated into routine care. In China, developers should prioritize WeChat-based platforms and culturally tailored features to address social pressures such as the ritual of “offering a cigarette.” A stratified delivery model is recommended: interactive apps for users under 40, and voice or SMS services for older adults.

## 1. Introduction

Tobacco dependence is a chronic and recurrent disease, recognized as a major preventable cause of death globally [1] and a significant threat to the health of Chinese residents [2]. Cigarette smoke exposure triggers airway epithelial cell-derived exosomal signaling that promotes M1 macrophage polarization and pyroptosis, contributing to chronic obstructive pulmonary disease pathogenesis [3]. Despite the availability of effective behavioral and pharmacological interventions, traditional smoking cessation services encounter challenges such as low coverage, limited accessibility, and poor adherence [4]. Digital health interventions delivered via mobile phones, including short message services (SMS) and smartphone applications (Apps), and emerging conversational agents powered by artificial intelligence, present innovative solutions to these challenges. These interventions offer advantages such as high penetration rates, immediacy, low cost, and personalization [5]. Alongside these digital modalities, electronic cigarettes have been promoted by some as a harm-reduction alternative to combustible tobacco; however, a growing body of evidence indicates that e-cigarette aerosols contain carcinogenic constituents [6]. Recent years have witnessed exponential growth in related research, with multiple systematic reviews affirming their effectiveness [7,8]. However, while international evidence supports their efficacy, these interventions face obstacles when directly applied to the Chinese context. On one hand, the available applications vary in quality and lack effectiveness validation specific to the Chinese population. On the other hand, there remains an absence of a clear implementation pathway for integrating these interventions with the smoking cessation service system tailored to Chinese characteristics [9]. Moreover, the rapid evolution of digital technologies, including AI-driven chatbots and large language models, introduces both opportunities and challenges that require careful evaluation in terms of safety, privacy, and clinical oversight [10]. This study aims to systematically summarize the best evidence regarding the role, mechanisms, functions, and intervention strategies of mobile smoking cessation programs for adult non-pregnant smokers. Additionally, it seeks to provide recommendations that consider the specific circumstances in China, thereby offering valuable insights for the advancement of mobile smoking cessation initiatives in the country.

## 2. Methods

This evidence summary is reported according to the PRISMA recommendations, and the protocol was prospectively registered with the Centre for Evidence-Based Nursing, Fudan University (registration number: ES20269952).

### 2.1. Establishment of the Problem

The PIPOST model was employed to develop evidence-based problems: (1) Target population: adult smokers aged 18 years or older who are not pregnant; (2) Intervention measures: a mobile health smoking cessation initiative primarily utilizing smartphone applications, SMS services, and emerging digital tools such as AI-driven chatbots; (3) Professionals involved: clinical doctors, clinical nurses, and public health workers; (4) Outcomes assessed: smoking cessation rates (7-day point prevalence abstinence, sustained abstinencepoint smoking cessation rate and sustained smoking cessation rate), changes in smoking volume, and intervention participation rates; (5) Locations: medical institutions, communities, households, and online platforms; (6) Types of evidence: clinical decision-making, clinical practice guidelines, expert consensus, evidence summaries, systematic reviews, and high-quality randomized controlled trials.

### 2.2. Evidence Retrieval

According to the “6S” evidence pyramid model [11], a top-down search was conducted utilizing the following resources: UpToDate, BMJ Best Practice, JBI Centre for Evidence-Based Health Care, Guidelines International Network, WHO Guidelines Database, Scottish Inter-Hospital Guidelines Website, AHRQ National Guidelines Centre, MedLink, Cochrane Library, PubMed, Web of Science, Embase, Sinomed, China National Knowledge Infrastructure (CNKI), Wanfang Database, and the Chinese Medical Journal Full-text Database. Additionally, a manual search was performed for the United States Preventive Services Task Force (USPSTF) and the National Institute for Health and Care Excellence (NICE) of the United Kingdom.

The search terms included: adults, chainsmokers, tobacco users, mHealth, mobile health, digital health, smartphone, mobile phone, cellphone, text messaging, SMS, messaging intervention, and mobile app/application-based; smoking cessation, quit smoking, tobacco cessation, and smoking abstinence. Given the current state of digital healthcare and smoking cessation interventions, as well as the rapid evolution of related research, the search timeframe was limited to the past 10 years, specifically from January 2016 to December 2025. To ensure completeness and incorporate recently published high-quality evidence, the search was updated to 31 August 2026. The complete, database-specific search strategies, including all Boolean operators, MeSH/Emtree terms, filters, and the date of the final search for each database, are provided in Supplementary Table S1 to ensure full reproducibility.

### 2.3. Literature Screening and Quality Evaluation

Literature screening, data extraction, and quality evaluation were conducted independently by two researchers trained in systematic evidence-based methods. The inclusion criteria were as follows: ① The research subjects were adult non-pregnant smokers (≥18 years old); ② The intervention measures consisted of mobile health interventions primarily delivered through SMS, applications, or AI-driven conversational agents. The exclusion criteria included: ① Non-Chinese and non-English literature; ② Duplicate publications or inability to obtain the full text; ③ Studies with excessively low quality evaluations, defined as studies failing to meet at least 50% of the applicable quality assessment domain criteria (e.g., AGREE II domain score < 30% for guidelines, or AMSTAR 2 critical flaws resulting in a “critically low” rating).

To manage overlap among included systematic reviews, we adopted a citation matrix approach. When the same primary study appeared in multiple systematic reviews, we retained the most recent, highest-quality review for evidence extraction and used other reviews for cross-validation of findings. A study overlap table is provided in Supplementary Table S2.

The included clinical decisions represent the highest level of evidence in the “6S” pyramid, characterized by high evidence quality, thus obviating the need for further quality evaluation. The AGREE II tool was employed to assess guideline quality [12], while the quality of expert consensus was evaluated using the expert consensus and opinion text evaluation tool from the JBI Evidence-Based Health Care Center [13]. The AMSTAR 2 scale was utilized to evaluate the quality [14] of the systematic reviews. The quality of authoritative reports was assessed using the “National Statistical Quality Assurance Framework (2021)” [15]. Finally, the quality of randomized controlled trials (RCTs) was evaluated using the Cochrane Risk of Bias Assessment tool, RoB 2.0 [16].

### 2.4. Evidence Synthesis and Grading

The evidence summary and classification are conducted through a thorough review and analysis of the literature, extracting pertinent clinical evidence for synthesis. The summary adheres to the following principles: when evidence from various sources is consistent or complementary, it is consolidated. In cases of conflicting evidence, priority is given to evidence-based findings, high-quality studies, and the most recently published data. When a recommendation item was derived from multiple sources (e.g., a guideline plus a systematic review), the final evidence level was determined by taking the highest level among the contributing sources, provided that at least two high-quality sources (Level 1 or 2) supported the recommendation. If only lower-level sources (Level 3–5) were available, the final level was capped at Level 3 to reflect the lower certainty. This rule ensures that recommendations are not over-graded based on a single high-level source when corroborating evidence is weak, and conversely, that a strong consensus across multiple moderate-level sources is appropriately recognized.

The classification of evidence follows these guidelines: included guidelines retain their original classification systems, while the remaining literature utilizes the JBI Evidence Pre-Classification and Evidence Recommendation Level System (2014Edition) [17], categorizing evidence levels from high to low across five grades. The strength of evidence recommendations is determined in accordance with the principles outlined in the JBI evidence recommendation strength classification.

## 3. Results

### 3.1. General Information of the Included Literature

**Figure 1:**
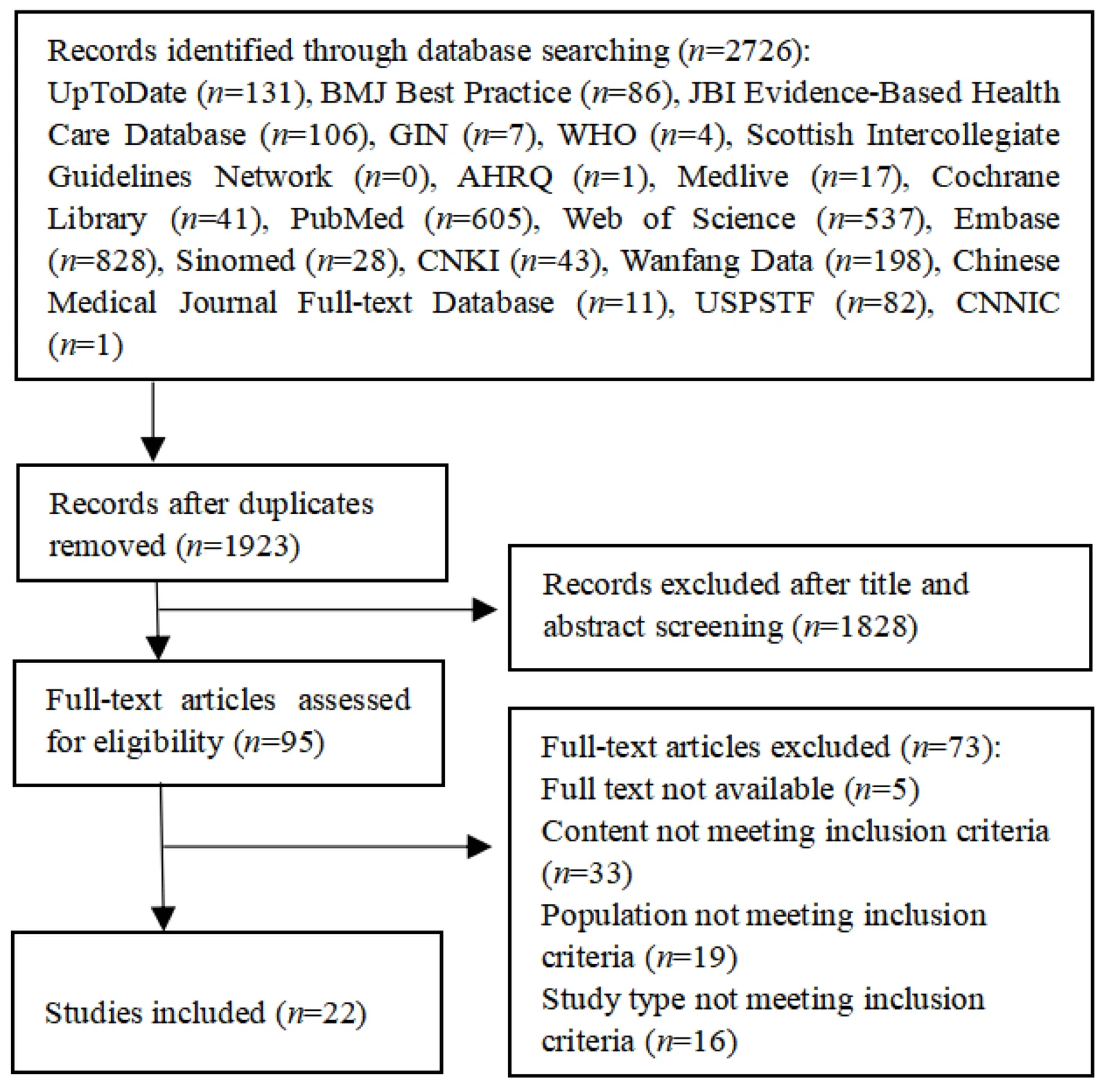
PRISMA flow diagram of study selection.

A preliminary search identified 2726 documents. Following the removal of duplicates, 1923 documents remained. After reviewing the titles and abstracts, 1828 articles were excluded, and 95 full-text articles were assessed for eligibility. Of these, 73 were excluded with reasons (see Supplementary Table S3 for the full list of excluded studies and reasons), and 22 were initially included. The literature screening process is illustrated in Figure 1. The fundamental characteristics of the included literature are presented in Table 1.

### 3.2. Literature Quality Results

This study included a total of five guidelines. Quality evaluation was conducted using AGREE II, revealing that the recommendation levels were consistently high. Specific evaluations are presented in Table 2. Additionally, the study incorporated one expert consensus, assessed with the JBI expert consensus evaluation tool, which met quality standards across six dimensions, including clear sources of viewpoints, influential experts, conclusions grounded in analytical results, and comprehensive engagement with existing literature. The overall quality was deemed relatively high. Furthermore, 12 systematic reviews were included, with quality assessed using the AMSTAR 2 scale, which indicated that the recommendation levels of all included guidelines were also relatively high. For detailed evaluations, please refer to Table 3. The study featured 1 randomized controlled trial (RCT), evaluated using the Cochrane Risk of Bias Assessment Tool. With the exception of item 4 (blinding of participants and personnel), which was rated as having some concerns, all other items across the RCT were classified as low risk and of high quality. Lastly, the study included one authoritative statistical report, evaluated according to the “National Statistical Quality Assurance Framework (2021)” [38] While the evaluation result for dimension 6, economic, was rated as good, all other dimensions received excellent ratings, indicating a relatively high quality.

### 3.3. Evidence Description and Synthesis

The study encompassed a total of 22 papers, which included 2 clinical decisions, 5 guidelines, 1 expert consensus, 12 systematic evaluations, 1 randomized controlled trial (RCT), and 1 authoritative statistical report. A summary of 28 pieces of best evidence was organized into five themes, as outlined in Table 4.

## 4. Discussion

### 4.1. Role Positioning of Mobile Smoking Cessation

Evidence 1 to 3 demonstrates that mobile smoking cessation, as a critical component of the tobacco control service system, is not designed to replace traditional smoking cessation services; rather, it serves as an effective extension and valuable supplement to them. The two approaches complement one another. From the perspective of intervention levels, mobile smoking cessation services provide a wide-reaching, cost-effective, convenient, and accessible entry point for individuals seeking to quit smoking, while also offering a continuous support platform. These services eliminate the temporal and spatial barriers associated with traditional smoking cessation methods. Functionally, mobile smoking cessation services act as a catalyst for behavioral change. By leveraging effective behavioral change theories, they enhance smokers’ intrinsic motivation and external capabilities for behavioral change through reminders, monitoring, and encouragement, thereby potentially increasing the success rate of smoking cessation [39]. The World Health Organization (WHO) has recognized mobile smoking cessation as part of the best practice intervention measures within the MPOWER framework [5]. From a social value perspective, traditional smoking cessation services exhibit limited reach, primarily serving urban populations. In contrast, mobile smoking cessation programs can effectively access remote and rural areas, as well as individuals who may be hesitant to seek face-to-face assistance due to social factors. Notably, the number of rural Internet users in China has reached 301 million [37]. This demographic deserves equitable access to smoking cessation services. Mobile smoking cessation initiatives have significantly improved the fairness and inclusivity of public health service provision. Consequently, the WHO and other organizations advocate for the integration of mobile smoking cessation services into national and local smoking cessation systems, positioning them as standard options for all adult smokers who wish to quit [20,21].

### 4.2. The Core of Improving the Effectiveness of Mobile Smoking Cessation Services

#### 4.2.1. Core Mechanism

Evidence 4 and 5 suggest that the fundamental principle of mobile smoking cessation lies in the comprehensive integration of established behavioral psychology theories with the inherent advantages of mobile devices, such as portability, real-time functionality, and personalization. Notably, mobile smoking cessation services that utilize applications effectively address the significant support gap present in traditional cessation methods, particularly when users are disconnected from in-person support. Through the use of preset information libraries, active push notifications, and tailored trigger scenarios, mobile smoking cessation programs can deliver continuous and precise intervention support during critical moments. This approach enables users to manage the onset of smoking cravings effectively and reinforces their efforts to quit smoking [40].

#### 4.2.2. Functional Module

In the core functional module Settings of the application, Evidence 6–11 indicates that features such as automatic tracking, personalized matching, health education, withdrawal symptom management, and social support are effective components of mobile smoking cessation applications. Brin et al. conducted five rounds of usability tests, continuously optimizing the visualization of smoking behavior based on user feedback, which included charts and progress displays. They concluded that presenting data visually is a fundamental function of the smoking cessation application [41]. Although Schiek, H. et al. reported that more frequent progress tracking was associated with smoking cessation (OR = 1.02, 95% CI: 1.00–1.03), the modest effect size raises questions about its clinical utility. Caution is warranted, however, as the unit of analysis for “frequency of visits” was not specified, limiting cross-study comparability and practical application [42]. Personalized matching moves away from a “one-size-fits-all” approach, offering tailored intervention support and ongoing services that consider users’ specific circumstances, including their level of nicotine dependence, smoking habits, stage of quitting, and relevant scenarios. Wu, Y.S. et al. [43] provided simple smoking cessation advice and facilitated active referrals to smoking cessation services while employing more interactive and adaptive instant messaging applications on smartphones, which included personalized services. Although the intervention did not significantly enhance the smoking cessation rate, it did markedly increase the frequency of utilization of smoking cessation services. Research indicates that participants who engaged with the app more frequently and took part in a wider array of activities were more likely to sustain a smoking cessation state at weeks 6 and 12 [44]. The primary function of health education is to reinforce motivation and reshape cognition. Studies have shown that, in comparison to self-help materials and smoking cessation apps, health education and multimodal interventions yield greater benefits for smoking cessation. Furthermore, health education and motivational interviewing demonstrate significant effects on short-term smoking cessation outcomes [45].

Adherence and engagement are critical determinants of mobile smoking cessation effectiveness. Evidence from included studies indicates that retention rates vary substantially: Bricker et al. reported 3-month, 6-month, and 12-month follow-up data retention rates of 86.7%, 88.4%, and 87.2%, respectively, in a trial comparing an acceptance and commitment therapy-based app with a guideline-based app [36]. Regarding intervention features, Barroso-Hurtado et al. found that most smoking cessation apps included self-tracking and quit plan setting, with apps combined with face-to-face interventions additionally incorporating carbon monoxide measurement [27]. Schiek et al. conducted a pilot study of a novel program integrating app-based behavioral therapy and an electronic cigarette, reporting 7-day point prevalence abstinence rates of 39.4% at 12 weeks and 32.4% at 24 weeks, with most participants rating the program as highly or moderately acceptable [42]. Message completion and engagement metrics vary across intervention modalities; Whittaker et al. found moderate-certainty evidence that text messaging interventions improve quit rates compared with minimal support (RR 1.59, 95% CI 1.09–2.33), while evidence comparing smartphone apps with less intensive support was of very low certainty (RR 1.00, 95% CI 0.66–1.52) [8]. A detailed framework synthesizing adherence and engagement indicators—including definitions, measurement approaches, and evidence summaries—is provided in Supplementary Table S6.

#### 4.2.3. Intervention Measures

Awareness of mobile smoking cessation services, a novel approach to quitting smoking, remains low; thus, initial promotional efforts must be intensified. Evidence 12 indicates that incorporating mobile smoking cessation information on cigarette packaging, along with increased exposure through mass media, can enhance both awareness and accessibility. This strategy offers a new alternative for smokers who wish to quit but find traditional cessation services inconvenient or unappealing [5]. Although mobile smoking cessation interventions differ from traditional models in terms of delivery methods and service formats, the underlying psychological motivations, behavioral change patterns, and core processes among smokers exhibit significant consistency. Consequently, evidence 13, 14, 21, and 22 can draw upon and adhere to the established frameworks and normative standards of traditional smoking cessation guidelines regarding pre-cessation assessments, intervention phase delineation, follow-up management, and outcome evaluation. Evidence 15 indicates that for smokers in the preparation stage, the combination of behavioral therapy and pharmacotherapy yields optimal outcomes, with mobile online intervention serving as a critical component of behavioral therapy [18]. Evidence 16 suggests that the duration of mobile smoking cessation interventions should be no less than six months, supplemented by long-term follow-up monitoring to reinforce the intervention’s effectiveness. The frequency of information dissemination should adhere to a high-frequency approach during the initial phase, transitioning to low-frequency maintenance in the later stages [21]. A recent multicenter cluster randomized clinical trial by Zahid, M. et al. (2026) [46] was identified in the updated search but was excluded from the formal evidence synthesis because its study population comprised exclusively patients with drug-sensitive pulmonary tuberculosis, a clinically distinct group that does not meet the PIPOST-defined target population of “adult non-pregnant smokers.” The trial enrolled daily smokers aged 15 years or older who had been diagnosed with drug-sensitive pulmonary TB within the preceding 4 weeks; TB diagnosis was a core eligibility criterion rather than an incidental comorbidity. The intervention was explicitly designed to leverage the TB diagnosis as a”teachable moment”, and the primary outcomes included TB treatment adherence, treatment success, and mortality—outcomes specific to the TB care context and not applicable to general adult smokers. Including this trial would therefore violate the pre-specified population criteria and introduce substantial clinical heterogeneity. This study is cited here only as supporting contextual evidence. Evidence 16–20 elucidates the personalized realization pathway of mobile smoking cessation interventions at the implementation level. This indicates that mobile health technology can utilize digital platforms to concurrently achieve dual personalization in both intervention stages and content. It can dynamically modify the intensity of interventions and push strategies according to the behavioral change stage, the level of tobacco dependence, and the real-time withdrawal status of smokers. Such adaptability significantly enhances the accuracy and adherence to the intervention. Numerous systematic reviews and meta-analyses have established that personalized design is a critical factor in attaining stable and long-term cessation outcomes in mobile smoking cessation interventions [28,47,48]. The intervention content prioritizes cultural adaptability. By considering the variations in cultural backgrounds, values, behavioral customs, and health perceptions among diverse populations, cultural elements are intricately woven into the entire process of plan design, content delivery, and function optimization. This approach ensures that the intervention measures align more closely with the psychological needs and behavioral traits of the target group, thereby addressing the barriers to smoking cessation interventions that arise from cultural differences [32,49]. Mobile smoking cessation interventions should prioritize the adaptation of content to specific temporal milestones. For example, during public health observances that highlight the dangers of tobacco and promote health, such as World No Tobacco Day and World Heart Day, it is essential to deliver targeted intervention content focused on smoking cessation themes. This strategic timing can effectively enhance smokers’ health awareness and emotional engagement with cessation efforts, thereby fostering the development and reinforcement of their short-term motivation to quit. Ultimately, this approach may facilitate the transition from short-term motivation to sustained smoking cessation and withdrawal behaviors, thereby improving both the immediate effectiveness and long-term cessation rates associated with mobile smoking cessation interventions [50].

### 4.3. Feasibility Analysis of Promoting Mobile Smoking Cessation in China

Each year, more than one million individuals in mainland China die as a result of smoking. This figure is projected to rise to two million by 2030 and reach three million by 2050 [51]. In 2024, the annual smoking rate among adults in China was 23.2% [52]. Although 18% of smokers intend to quit within the next 12 months, the uneven distribution of medical resources, limited access to smoking cessation medications, and insufficient specialized training for healthcare providers hinder the availability of effective smoking cessation services for most smokers [53]. China possesses the largest mobile Internet user base globally. The extensive penetration of smartphones has established a robust hardware foundation for the advancement of mobile smoking cessation services [30]. A realistic assessment study of Chinese smokers has confirmed that they generally perceive smoking cessation apps as valuable tools. Notably, smokers who are motivated to quit are more inclined to attempt and utilize these digital intervention methods [54]. Furthermore, the effectiveness of mobile smoking cessation interventions in China has shown promising preliminary evidence from high-quality local studies. The smartphone application “Smokefree Life,” which is based on cognitive behavioral therapy, can enhance the biochemically verified six-month continuous smoking cessation rate among adult smokers in China to 2.52 times that of the control group (6.50% vs. 2.58%) [55]. From a policy standpoint, the Chinese government maintains a favorable attitude toward digital health technology, thereby fostering a conducive policy environment for the advancement of mobile smoking cessation initiatives. In 2025, the State Council’s “Opinions on Deeply Implementing the ‘Artificial Intelligence Plus’ Action” called for the exploration and promotion of high-level health assistants that are accessible to all, while systematically advancing the application of artificial intelligence in areas such as auxiliary diagnosis, treatment, and health management [56]. The “Opinions on Promoting and Regulating the Application and Development of ‘Artificial Intelligence + Medical and Health Care’”, issued by five departments, including the National Health Commission in 2025, clearly states that by 2027, several high-quality datasets and specialized large models in the health and medical sectors will be established. In the “Grassroots Application” [57] section, the document proposes the implementation of intelligent services, such as chronic disease screening and personalized intervention, aimed at creating a new intelligent service model for chronic disease management at the grassroots level. In conclusion, given the substantial population of smokers in China and the uneven distribution of public health resources, mobile smoking cessation emerges as a crucial and inclusive intervention for addressing tobacco control. This approach offers significant advantages, including a high reach rate and low costs. Furthermore, its promotion in China benefits from an established user base and demonstrated local efficacy, alongside a supportive policy environment. As new technologies, such as AI, are integrated and intervention models are refined, mobile smoking cessation is poised to become an essential component of China’s tobacco control initiatives, significantly contributing to the realization of the “Healthy China” goal.

### 4.4. Thoughts and Suggestions in the Chinese Context

A survey indicates that the smoking rate among adults aged 15 and older in China is 23.2% [58], surpassing the global average of 16.4% [59]. The age distribution of both the smoking rate and the quantity of cigarettes consumed exhibits a distinct “inverted U-shaped” trend. Specifically, the smoking rate is 19.0% for individuals aged 15–29, peaks at 27.2% among those aged 40–49, and subsequently declines with advancing age. The average consumption for smokers aged 15–29 is 12.8 cigarettes per day, reaching a maximum of 19.1 cigarettes per day for the middle-aged and elderly population aged 50–69, before gradually decreasing with age [52]. However, it should be noted that the suggestion in Evidence 16 regarding age-based stratification (under 40 vs. over 40) is a contextual hypothesis derived from observational usability studies and general smoking cessation guidance, rather than a direct evidence-based recommendation from comparative trials [31]. This demographic characteristic imposes practical limitations on the promotion and implementation of mobile smoking cessation services. According to the “52nd Statistical Report on the Development of China’s Internet,” middle-aged and elderly populations prefer straightforward applications that do not necessitate complex operations, such as plain text messages, telephone hotlines, and voice interactions [37]. Consequently, when implementing mobile smoking cessation initiatives in China, it is advisable to consider stratified interventions based on age, but this should be viewed as a starting point rather than a rigid rule. For individuals under 40, who are generally more digitally literate, it is beneficial to enhance lightweight and highly interactive mobile interventions, such as apps and interactive text messages. Conversely, for the primary smoking demographic over 40, who may have lower digital proficiency, it is essential to develop minimalist mobile tools that align with their usage habits, including SMS reminders, mini-programs, and voice assistance, thereby reducing the operational threshold. Crucially, future implementation should move beyond simple age stratification toward data-driven, multidimensional user profiling that incorporates digital literacy, device usage history, baseline nicotine dependence, past app engagement, and socioeconomic factors, using unsupervised learning or recommender systems to dynamically allocate users to the most appropriate intervention modality. Simultaneously, leveraging the lightweight, highly shareable, and easily digestible nature of short videos can facilitate the dissemination of smoking cessation education, intervention tutorials, and interactive content to the broader population, thereby lowering barriers to smoking cessation interventions and expanding the reach of mobile smoking cessation efforts.

In Chinese social settings, the practice of “offering cigarettes” among smokers is deeply ingrained. This behavior has evolved beyond mere physiological needs into a significant social exchange ritual. In this context, declining an offer of cigarettes is frequently perceived as carrying negative social implications, often interpreted by the other party as a deliberate attempt to distance oneself from interpersonal relationships or even as a subtle rejection of one’s social status. Consequently, this cultural custom exerts considerable social pressure on smokers, particularly those who wish to quit [60]. In light of the distinctive “smoking respect” culture and the social pressure it imposes on individuals seeking to quit smoking, it is recommended that a systematic intervention strategy be developed along two dimensions: macro-environmental reshaping and micro-individual empowerment. To effectively tackle the pressure associated with “offering cigarettes” in Chinese social contexts, a coordinated approach should be implemented at both the macro cultural level and the micro skill development level.

At the macro level, it is essential to address cultural deviations and reconstruct social norms. Utilizing mobile smoking cessation platforms and integrating emerging communication channels, such as short videos and social media, the contemporary social concept of “refusing cigarettes does not harm relationships, and quitting smoking is commendable” should be widely promoted. The notions of declining cigarette offers and refraining from encouraging others to smoke should be clearly defined and established as the civilized etiquette of the new era, thereby gradually diminishing the symbolic significance of tobacco in interpersonal interactions. Concurrently, a social support ecosystem encompassing various scenarios should be developed. This includes establishing an online interaction mechanism among smokers, their families, colleagues, and healthcare providers, which can effectively alleviate the pressures individuals encounter in real social contexts. Community-based smoking cessation contracts and the promotion of group consensus can further enhance this support system. Furthermore, it is essential to integrate policy advocacy with scenario-based tools effectively. This integration should enhance the dissemination and education of smoking control policies in public spaces via online platforms. Additionally, mobile smoking cessation services should be linked to the certification systems for smoke-free units, smoke-free families, and smoke-free social interactions. Such measures aim to guide public behavior through positive incentives, thereby replacing passive social pressure.

At the micro level, it is crucial to improve the practicality of mobile smoking cessation tools. These tools should include a standardized and emotionally intelligent library of no-smoking phrases, featuring pre-set gentle, respectful, and non-irritating expressions to assist users in managing situations where smoking is offered. This approach will help minimize social concerns to the greatest extent possible. Concurrently, it is important to develop a simulation training function for anti-smoking scenarios, incorporating role-playing desensitization exercises through short videos and interactive modules. These initiatives are hypothesized to effectively enhance the on-the-spot response capabilities and psychological resilience of individuals seeking to quit smoking and should be evaluated in future research.

### 4.5. Barriers and Facilitators to Implementation in China

To guide local adaptation, we summarize key implementation barriers and facilitators in Table 5, drawing from the synthesized evidence and China-specific contextual analysis. This framework can inform policy-making, intervention design, and resource allocation for mobile smoking cessation programs in China.

### 4.6. Implications for AI and Emerging Technologies

Evidence 23 highlights the potential of AI-driven conversational agents and passive sensing for smoking cessation [65]. Large language models (LLMs) offer scalable, 24/7 personalized support through natural language interactions, potentially lowering barriers to help-seeking. However, several critical issues must be addressed before deployment in clinical or public health settings. First, misinformation and hallucination risks are non-trivial; LLMs may generate inaccurate or inconsistent cessation advice, particularly regarding medication dosing or withdrawal management. Second, data privacy and security are paramount, as sensitive health information is shared through conversational interfaces. Third, the absence of clinical oversight raises accountability concerns; human-in-the-loop safeguards (e.g., escalation to human counselors for high-risk situations) are essential. Fourth, cultural and linguistic adaptation of LLMs for Chinese populations remains underdeveloped. Regulatory frameworks, such as those outlined in China’s “AI + Healthcare” guidelines [57], must evolve to address these challenges. We recommend pilot studies evaluating AI chatbot safety, efficacy, and user acceptance in controlled settings prior to wider implementation.

### 4.7. Limitations

Several limitations should be acknowledged. First, this evidence summary primarily relies on secondary sources (guidelines, systematic reviews, expert consensus), with only 1 directly included RCT. This design introduces the possibility of overlap and double-counting of primary studies across the included systematic reviews. We attempted to mitigate this through a citation matrix approach (Supplementary Table S2), but some degree of duplication is inherent in this type of synthesis. Second, the language restriction to Chinese and English may have introduced language bias, potentially excluding relevant studies published in other languages. Third, we did not systematically search grey literature (e.g., conference proceedings, unpublished reports), which may contain relevant implementation evidence. Fourth, the rapid evolution of mobile technologies means that some evidence, particularly on specific app features or AI tools, may become outdated quickly; the findings should be interpreted with this temporal limitation in mind. Fifth, while we updated the search to August 2026 to capture recent high-quality studies, the heterogeneity in intervention types, cessation definitions, and follow-up durations across the included reviews limits the precision of our synthesis. Finally, the China-specific recommendations, particularly those related to cultural adaptation (e.g., “offering cigarettes”) and age stratification, are based on contextual analysis and observational insights rather than direct comparative evidence; they should be considered as hypotheses for future implementation research rather than firm practice mandates.

## 5. Conclusions

This study employed an evidence-based approach to systematically integrate the best practices for mobile smoking cessation interventions targeting adult non-pregnant smokers. It examined their roles, mechanisms, functions, and intervention measures. In light of the current prevalence and cultural characteristics of smoking in China, the study proposed localized implementation recommendations, thereby providing both theoretical references and a practical foundation for the advancement of mobile smoking cessation initiatives in the country. Nonetheless, the existing evidence base primarily derives from high-quality international research. Although domestic perspectives have been included to enhance this body of work, robust efficacy evidence specific to the Chinese population remains inadequate. Future research should prioritize: (1) validating the effectiveness of behavior change techniques tailored to the psychological characteristics of Chinese culture; (2) analyzing the mechanisms underlying the integrated implementation framework for mobile smoking cessation; and (3) assessing the cost-effectiveness and sustainability of this framework in real-world medical settings. (4) developing and validating AI-driven personalized cessation tools with appropriate safeguards; and (5) conducting implementation science studies to evaluate the uptake, fidelity, and scalability of mobile cessation interventions across diverse Chinese settings, particularly addressing the digital divide. To achieve these objectives, it is imperative for public health departments, medical institutions, academia, and industry to collaborate in developing a mobile smoking cessation implementation strategy that aligns with China’s national conditions.

## Figures and Tables

**Table 1:** Basic characteristics of the included literature.

Literature	Year	Source	Type of Literature	Topic of Literature
Park, E.R. et al. [18]	2025	UpToDate	Clinical Decision	Behavioral therapy for smoking cessation
Rigotti, N.A. et al. [19]	2025	UpToDate	Clinical Decision	Overview of adult smoking cessation management
NICE [20]	2023	NICE	Guideline	Preventing smoking initiation, promoting smoking cessation, and treating tobacco dependence
USPSTF [21]	2021	AHRQ-NGC	Guideline	Smoking cessation interventions for adults and pregnant women
WHO [5]	2019	WHO	Guideline	2019 Global Tobacco Epidemic Report: Offering help for quitting tobacco
NCCN [22]	2025	Medlive	Guideline	NCCN Clinical Practice Guidelines: Smoking Cessation section
NHFPC [23]	2015	Medlive	Guideline	Chinese Clinical Smoking Cessation Guideline
Cheung, K.L. et al. [24]	2017	PubMed	Expert Consensus	How to evaluate smoking cessation intervention effects
Taylor, G.M.J. et al. [25]	2017	Cochrane	Systematic Review	Internet-based smoking cessation interventions
Chu, S. et al. [26]	2026	PubMed	Systematic Review	Effectiveness of smartphone apps alone versus combined with traditional interventions for smoking cessation
Barroso-Hurtado, M. et al. [27]	2021	Web of Science	Systematic Review	Forms, effects, and functions of smoking cessation apps: a systematic review
Li, S. et al. [28]	2025	Cochrane	Systematic Review	Digital smoking cessation intervention effects classified by type and method
Whittaker, R. et al. [8]	2019	PubMed	Systematic Review	SMS and app-based smoking cessation interventions
Ho Imran Ho, D.S. et al. [29]	2025	PubMed	Systematic Review	The influence of game incentive strategies on quitting smoking
Zhou, Y.H. et al. [30]	2019	PubMed	Systematic Review	Effectiveness of interventions to reduce secondhand smoke exposure from parental smoking in Chinese families
Shah, A. et al. [31]	2020	PubMed	Systematic Review	Personalized web-based smoking cessation interventions and reducing loss to follow-up
Leinberger-Jabari, A. et al. [32]	2024	Web of Science	Systematic Review	Effectiveness of culturally adapted smoking cessation interventions for reducing or ceasing combustible tobacco use
Alam, N.B. et al. [33]	2026	PubMed	Systematic Review	Digital phenotyping for predicting mood and relapse
Wilson, S. et al. [34]	2024	PubMed	Systematic Review	Strategies to Promote Digital Health Equity
Mohamed ElshikhIdris, M.A. et al. [35]	2026	PubMed	Systematic Review	AI chatbots for nursing and health interventions
Bricker, J.B. [36]	2020	PubMed	RCT	Effectiveness of smartphone app-assisted smoking cessation
CNNIC [37]	2023	CNNIC	Authoritative Statistical Report	The 52nd China Statistical Report on Internet Development

**Table 2:** Results of the quality assessment of included guidelines.

Included Literature	Standardized Percentage by Domain (%)						Number of Domains with ≥60%	Number of Domains with ≥30%	Recommendation Level
	Scope & Purpose	Stakeholder Involvement	Rigor of Development	Clarity of Presentation	Applicability	Editorial Independence			
NICE	96.30	94.44	96.30	98.15	92.59	94.44	6	6	A
USPSTF	94.44	92.59	94.44	96.30	90.74	88.89	6	6	A
WHO	90.74	88.89	92.59	94.44	88.89	90.74	6	6	A
NCCN	98.15	93.06	91.76	97.22	75.00	72.92	6	6	A
NHFPC	96.30	79.17	46.88	97.22	62.50	50.00	4	6	A

**Table 3:** Quality assessment results of included systematic reviews.

Assessment Item	Taylor, G.M.J. et al. [25]	Chu, S. et al. [26]	Hurtado, M. et al. [27]	Li, S. et al. [28]	Whittaker, R. et al. [8]	Ho Imran Ho, D.S. et al. [29]	Zhou, Y.H. et al. [30]	Shah, A. et al. [31]	Leinberger-Jabari, A. et al. [32]	Alam, N.B. et al. [33]	Mohamed ElshikhIdris, M.A. et al. [35]	Wilson, S. et al. [34]
(1) Did the research questions and inclusion criteria include the PICO elements?	Yes	Yes	Yes	Yes	Yes	Yes	Yes	Yes	Yes	Yes	Yes	Yes
(2) Was the systematic review methodology stated before conducting the review, and were any deviations from the methodology explained?	Yes	Yes	Yes	Yes	Yes	Yes	Partial	Partial	Yes	Yes	Partial	Yes
(3) Were the types of studies included in the literature specified?	Yes	Yes	Yes	Yes	Yes	Yes	Yes	Yes	Yes	Yes	Yes	Yes
(4) Was a comprehensive search strategy employed?	Yes	Yes	Yes	Yes	Yes	Yes	Yes	Yes	Yes	Partial	Partial	Yes
(5) Was study selection performed by two reviewers independently?	Yes	Yes	Yes	Yes	Yes	Yes	Yes	Yes	Yes	Yes	Yes	Yes
(6) Was data extraction performed by two reviewers independently?	Yes	Yes	Yes	Yes	Yes	Yes	Yes	Yes	Yes	Yes	Yes	Yes
(7) Was a list of excluded studies provided with reasons for exclusion?	Yes	Partial	Yes	Yes	Yes	Yes	Partial	Yes	Yes	Partial	Partial	Partial
(8) Were the included studies described in detail?	Yes	Yes	Yes	Yes	Yes	Yes	Yes	Yes	Yes	Yes	Yes	Yes
(9) Was an appropriate tool used to assess the risk of bias for each included study?	Yes	Yes	Yes	Yes	Yes	Yes	Yes	Yes	Yes	Yes	Yes	Yes
(10) Were the funding sources of each included study reported?	Partial	Yes	No	No	Yes	No	No	No	No	No	No	No
(11) Was an appropriate statistical method used to synthesize the study results?	Yes	Yes	N/A	Yes	Yes	Yes	N/A	Yes	Yes	N/A	N/A	N/A
(12) Was the risk of bias of each included study and its potential impact on the meta-analysis or other evidence synthesis results assessed?	Yes	Yes	Yes	Yes	Yes	Yes	N/A	Yes	Yes	N/A	N/A	N/A
(13) When interpreting or discussing each study result, was the risk of bias of the included studies taken into account?	Yes	Yes	Yes	Yes	Yes	Yes	Yes	Yes	Yes	Yes	Yes	Yes
(14) Was any heterogeneity in the study results reasonably explained and discussed?	Yes	Yes	Yes	Yes	Yes	Yes	Yes	Yes	Yes	Yes	Yes	Yes
(15) If quantitative synthesis was performed, was publication bias adequately investigated and its potential impact on the results discussed?	Yes	N/A	N/A	Yes	Yes	Yes	Yes	Yes	Yes	N/A	N/A	N/A
(16) Were all sources of potential conflicts of interest reported, including any funding received for producing the systematic review?	No	Yes	Yes	Yes	Yes	Yes	Yes	Yes	Yes	Yes	Yes	Yes
Quality Rating	High	High	Moderate	High	High	High	Moderate	High	High	Moderate	Moderate	Moderate

N/A: not applicable.

**Table 4:** Best evidence of mobile smoking cessation interventions for non-pregnant adult smokers.

Evidence Category	Best Evidence Content	Evidence Level	Recommendation Strength	Evidence Type
Role Positioning	**1.** Evidence-based mobile cessation interventions, such as text messaging and applications, should be incorporated into national and local cessation service systems as a standard option available to all adult smokers seeking to quit [20,21].	1	A	General cessation guideline + Mobile-specific evidence
	**2.** The integration of mobile interventions with first-line smoking cessation medications can yield a synergistic effect, resulting in a long-term smoking cessation rate that significantly surpasses that achieved through single interventions. The World Health Organization has identified mobile smoking cessation interventions as one of the “best practices” for promoting cessation efforts [25].	2	A	General cessation guideline
	**3.** Mobile-based interventions are capable of effectively engaging underserved populations—such as rural communities, low-income groups, and ethnic minorities—and thus represent a key strategy for mitigating inequities in the availability and utilization of tobacco cessation services [5].	1	A	Contextual recommendation
Core Mechanisms	**4.** It is essential to elucidate the theoretical driving forces and mechanisms involved. Intervention measures should be grounded in well-defined behavioral change theories, such as cognitive behavioral therapy, social cognitive theory, and acceptance and commitment therapy. Additionally, a clear pathway for the mechanism of action should be established [22,26].	1	A	Mobile-specific evidence
	**5.** To establish an effective mobile smoking cessation intervention, it is necessary to have a preset information database, an active push cycle and frequency, and trigger scenarios [18].	1	A	Mobile-specific evidence
Functional Modules	**6.** Establish automatic tracking to visualize check-in records. Applications and mini-programs should incorporate a feature that allows users to access their check-in records. Additionally, these platforms can provide real-time feedback on continuous check-in data through visual charts, thereby increasing user engagement and enhancing users’ self-efficacy [27].	2	B	Mobile-specific evidence
	**7.** Develop personalized matching by aligning intervention content with the user’s name, quit date, motivation for cessation, triggering scenarios, cultural background, and other relevant factors. Provide tailored support information that corresponds to the user’s current stage in the smoking cessation process [28].	4	A	Mobile-specific evidence
	**8.** Health education primarily aims to enhance motivation and reshape cognitive processes. It encompasses content modules that address motivation for smoking cessation, correct misconceptions, and manage the fear associated with quitting smoking [8].	2	A	General cessation guideline
	**9.** Withdrawal symptom management: It is necessary to integrate diverse and immediately available withdrawal reaction coping toolkits and set up online consultation services for smokers to choose from [8].	2	A	General cessation guideline
	**10.** Social support enables users to invite friends and family as support partners and to receive notifications regarding their progress. Users can share their experiences and receive encouragement from peers. Additionally, users can share their achievements on social media platforms [8].	2	A	General cessation guideline
	**11.** Avoiding gamification and incentive strategies is advisable. While incorporating elements like points, badges, challenges, and small material rewards can boost short-term task completion rates and user engagement, their long-term impact diminishes over time, necessitating ongoing optimization to sustain user engagement [29].	4	A	Mobile-specific evidence
Methods and Approaches	**12.** To promote smoking cessation, it is essential to print mobile cessation resources, including hotline numbers, SMS registration links, and application information, on cigarette packaging and in mass media advertisements. This approach aims to enhance public awareness and increase utilization rates of these resources [5].	1	A	General cessation guideline
	**13.** Tobacco Use Assessment: Perform a systematic evaluation utilizing the 5A method to inquire about and document tobacco use circumstances. Assess the individual’s readiness to quit smoking by explicitly asking about their willingness to do so within the next 30 days. Participants expressing a willingness to quit will proceed to the assistance stage, while those who are hesitant or unwilling will receive motivational interviews grounded in the 5R model [18].	1	A	General cessation guideline
	**14.** The stages of smoking cessation are categorized as follows: Pre-intention stage: individuals do not consider quitting smoking within the next six months; Intention stage: individuals contemplate quitting smoking within one to six months; Preparation stage: individuals plan to attempt quitting smoking within one month; Action stage: individuals actively try to quit smoking; Maintenance stage: individuals have successfully quit smoking for at least six months [19].	1	A	General cessation guideline
	**15.** Method Selection: For individuals prepared to quit smoking, the combination of behavioral therapy and medication is the most effective approach. It is essential that the selected behavioral therapy encourages all individuals ready to quit to engage in behavioral counseling programs. These counseling methods encompass face-to-face consultations, phone calls, text messages, online interactions, mobile applications, and various online resources. For those not yet ready to quit smoking, the 5R model can enhance motivation and increase the likelihood of future quit attempts [19].	1	A	General cessation guideline
	**16.** Establish a definitive start date for smoking cessation, accompanied by an intervention cycle lasting a minimum of six months. The dissemination of intervention information should adhere to a pattern characterized by high-frequency communication during the initial phase, transitioning to low-frequency maintenance in the subsequent stages [21,22].	1	A	General cessation guideline + Mobile-specific evidence
	**17.** A customized online intervention begins with screening and assessing individuals’ willingness and conditions to quit smoking. Subsequently, it tailors the frequency and content of information delivery based on individual characteristics, such as smoking quantity, motivation, and stage of readiness. Young individuals under the age of 40 are particularly well-suited for interventions delivered via apps and interactive SMS. (It is hypothesized that younger individuals under the age of 40 may be particularly well-suited for interventions delivered via apps and interactive SMS, but this age threshold is a contextual suggestion rather than a firm evidence-based rule. A data-driven, multidimensional user profiling approach (incorporating digital literacy, device usage patterns, and nicotine dependence) is preferred for personalized allocation.) [31].	3	B	Mobile-specific evidence
	**18.** Enhancing the cultural adaptability of intervention content necessitates the incorporation of localized languages, respect for cultural customs, integration of region-specific case studies, and the avoidance of sensitive topics to foster affinity and resonance. Additionally, it is crucial to align content with significant temporal milestones, such as promoting smoking cessation messages on World No Tobacco Day and World Heart Day. This strategic timing can evoke a deeper emotional response among smokers, thereby facilitating the transformation of short-term motivations into sustained cessation behaviors [32].	3	A	Mobile-specific evidence
	**19.** Ensure dynamic personalization and adaptability by adjusting the content, format, and timing of information delivery in response to real-time user behavior, emotional states, or environmental data. This approach aims to enhance the immediacy and precision of interventions [27].	2	B	Mobile-specific evidence
	**20.** Enhancing human–computer interaction and self-monitoring involves encouraging users to actively record data, including the frequency, timing, and contextual triggers of smoking urges. Providing immediate feedback and visualizing progress can substantially improve user engagement and self-efficacy. Additionally, the application should incorporate a positioning function that automatically delivers contextualized smoking cessation prompts upon recognizing the relevant scenario [36].	2	B	Mobile-specific evidence
	**21.** The follow-up time points of 1 month, 3 months, and 6 months represent globally standardized assessment intervals, complemented by a 12-month long-term withdrawal validation. The 6-month follow-up serves as the primary outcome measure, while the 1- to 3-month interval is designated for early predictions of short-term efficacy. The 12-month follow-up is employed to assess the long-term maintenance effect [24].	5	B	General cessation guideline
	**22.** Effect evaluation: Main indicators: 7-day point prevalence abstinence (PPA); Secondary indicators: sustained abstinence (>30 days), change in smoking volume [24].	5	B	General cessation guideline
	**23.** Passive sensing and predictive analytics: Emerging evidence supports the integration of passive smartphone sensor data (e.g., GPS location, accelerometer, screen time) to objectively identify emotional states and craving triggers without user burden. Machine learning algorithms applied to longitudinal user data can predict relapse risk trajectories and app attrition rates, enabling proactive just-in-time adaptive interventions (JITAIs). However, these approaches require rigorous validation, privacy safeguards, and clinical oversight before widespread deployment [33].	1	A	Contextual recommendation
Considerations Specific to China	**24.** Given the current state of China’s digital ecosystem, intervention design should prioritize development centered on super application platforms, such as WeChat mini-programs, to reduce barriers to user access. Additionally, it is essential to explore the interoperability of health data with platforms like Alipay and government applications [37].	4	B	Contextual recommendation
	**25.** The culturally specific challenges to smoking cessation in China primarily stem from the significant influence of family and social networks, which serve as the main barriers. Effective smoking cessation interventions must be integrated with targeted strategies that consider the cultural context of relationships (Guanxi), the concept of face, and collectivism. Emphasizing “quitting smoking for family health” as a central motivation can enhance these efforts, while reinforcing a family responsibility-driven model for smoking cessation [30].	3	B	Contextual recommendation
	**26.** Connect to the offline service system: The application must facilitate easy access to local smoking cessation clinic inquiries, provide an entry point for online consultations, and offer a one-click transfer feature to the national smoking cessation hotline at 400-888-5531 and the health hotline at 12320. This will create a seamless process of “online initial screening and intervention—offline in-depth service.” [23]	5	B	Contextual recommendation
	**27.** Addressing the digital divide: Implementation strategies must account for disparities in age, education, income, rural versus urban residence, smartphone access, and digital literacy. For populations with limited digital access, simplified voice-based services, SMS-only interventions, and integration with community health worker networks are recommended as complementary approaches [34].	4	B	Contextual recommendation
	**28.** AI-driven conversational agents: Large language model-based chatbots offer potential for scalable, 24/7 personalized cessation support. However, deployment must address risks of misinformation, hallucination, data privacy, and the absence of clinical oversight. Regulatory frameworks and human-in-the-loop safeguards are essential prior to implementation [35].	4	B	Contextual recommendation

**Table 5:** Barriers and facilitators to implementing mobile smoking cessation interventions in China.

Domain	Barriers	Facilitators
Accessibility [37]	– Digital divide: disparities in smartphone access, internet connectivity between urban and rural areas – Limited availability of high-quality, evidence-based apps in Chinese app stores	– High overall smartphone penetration rate (over 1 billion users) – Extensive reach of WeChat and other super-apps as delivery platforms
Digital Literacy [61]	– Low digital literacy among older adults (primary smoking demographic) – Limited proficiency in using complex app features	– Familiarity with basic mobile functions (SMS, voice calls, WeChat messaging) – Availability of simplified voice-based and SMS-only options
Healthcare Integration [23]	– Fragmented healthcare system, lack of clear referral pathways – Limited awareness among healthcare providers of mHealth cessation tools	– National policies supporting digital health and AI integration – Existing smoking cessation clinic network and national hotline (400-888-5531)
Cultural Adaptation [30,62]	– Social norms around “offering cigarettes” and “face” – Stigma associated with quitting or refusing cigarettes in social contexts	– Cultural emphasis on family health as a strong motivator – Collectivist values facilitating group-based cessation interventions
Privacy & Data Security [63]	– Concerns over personal health data collection and sharing – Lack of clear regulatory frameworks for mHealth data governance	– Existing data protection laws (e.g., Personal Information Protection Law) – Potential for anonymized data aggregation and encrypted storage
Adherence & Engagement [62,64]	– High attrition rates common in digital interventions – Low long-term engagement beyond initial weeks	– Personalization and tailoring features can boost engagement – Gamification elements (though limited long-term effects)
Cost & Sustainability	– Unclear cost-effectiveness compared to traditional interventions – Lack of reimbursement mechanisms for mHealth cessation services	– Low marginal cost for scaling digital interventions – Potential for integration into public health insurance packages
Regulatory Considerations [56,57]	– Absence of specific regulations for AI-driven chatbots in smoking cessation – Need for certification/quality standards for smoking cessation apps	– Supportive national policies for “AI + Healthcare” – Potential for regulatory pilot programs in digital health

## Data Availability

No new data were created or analyzed in this study. Data sharing is not applicable to this article.

## Supplementary Materials

The following supporting information can be downloaded at: https://www.mdpi.com/article/10.3390/healthcare14213644/s1, Table S1: Complete Database Search Strategies; Table S2a: Study Overlap Matrix Among Smoking Cessation Intervention Systematic Reviews; Table S2b: Systematic Reviews Addressing Cross-Cutting Digital Health Themes (No Overlap with Smoking Cessation RCTs); Table S3: List of Excluded Studies at Full-Text Screening with Reasons; Table S4: PRISMA 2020 Checklist; Table S5: Detailed Evidence Grading Rules; Table S6: Framework for Analyzing Intervention Adherence and Engagement; Table S7: Supplementary Table for China-Specific Barriers and Facilitators.
