# Supplementary material for: Evidence Synthesis of Mobile-Based Smoking Cessation Interventions for Adult Non-Pregnant Smokers and Practice Recommendations Based on the Chinese Context
Source: Healthcare (Basel). 2026 Oct 27;14(21):3644. doi: 10.3390/healthcare14213644 (PMC13763036; doi:10.3390/healthcare14213644)
Supplement: Supplementary file 1 [file healthcare-14-03644-s001.zip › healthcare-4496482-supplementary.pdf]

Supplementary Table S1: Complete Database Search Strategies

Search Time Range: January 2016-August 2026

Search Execution Date: September 1, 2026

Pubmed	((("adult"[MeSH Terms] OR "adults"[Title/Abstract] OR "adult smokers"[Title/Abstract]) AND ("smoking cessation"[MeSH Terms] OR "smoking cessation"[Title/Abstract] OR "quit smoking"[Title/Abstract] OR "tobacco cessation"[Title/Abstract] OR "smoking abstinence"[Title/Abstract])) AND (("mobile health"[Title/Abstract] OR "mHealth"[Title/Abstract] OR "digital health"[Title/Abstract] OR "smartphone"[MeSH Terms] OR "smartphone"[Title/Abstract] OR "mobile phone"[Title/Abstract] OR "cellphone"[Title/Abstract] OR "text messaging"[MeSH Terms] OR "SMS"[Title/Abstract] OR "mobile app"[Title/Abstract] OR "mobile application"[Title/Abstract] OR "chatbot"[Title/Abstract] OR "conversational agent"[Title/Abstract] OR "AI"[Title/Abstract] OR "artificial intelligence"[Title/Abstract])) AND ("2016/01/01"[Date - Publication] : "2026/08/31"[Date - Publication])) NOT ("pregnant"[Title/Abstract] OR "pregnancy"[MeSH Terms] OR "prenatal"[Title/Abstract] OR "perinatal"[Title/Abstract])
Cochrane Library	#1 MeSH descriptor: [Adult] explode all trees<br>#2 (adult or "adult smokers"):ti,ab,kw<br>#3 #1 or #2<br>#4 MeSH descriptor: [Smoking Cessation] explode all trees<br>#5 ("smoking cessation" or "quit smoking" or "tobacco cessation" or "smoking abstinence"):ti,ab,kw<br>#6 #4 or #5<br>#7 MeSH descriptor: [Smartphone] explode all trees<br>#8 MeSH descriptor: [Text Messaging] explode all trees<br>#9 ("mobile health" or mHealth or "digital health" or "mobile phone" or cellphone or SMS or "text messaging" or "mobile app" or "mobile application" or chatbot or "conversational agent" or "artificial intelligence"):ti,ab,kw<br>#10 #7 or #8 or #9<br>#11 #3 and #6 and #10 with Publication Year from 2015 to 2026<br>#12 (pregnant or pregnancy or prenatal or perinatal):ti,ab,kw<br>#13 #11 not #12
Web of science	((TS=(adult* OR "adult smokers" OR "adult smoking")) AND TS=("smoking cessation" OR "quit smoking" OR "tobacco cessation" OR "smoking abstinence")) AND TS=("mobile health" OR mHealth OR "digital health" OR smartphone* OR "mobile phone*" OR cellphone* OR SMS OR "text messaging" OR "mobile app*" OR "mobile application*" OR chatbot* OR "conversational agent*" OR AI OR "artificial intelligence")) NOT TS=(pregnant OR pregnancy OR prenatal OR perinatal)
Embase	('adult'/exp OR adult*:ti,ab OR 'adult smokers':ti,ab) AND ('smoking cessation'/exp OR 'smoking cessation':ti,ab OR 'quit smoking':ti,ab OR 'tobacco cessation':ti,ab OR 'smoking abstinence':ti,ab) AND ('mobile health'/exp OR

	'mhealth'/exp OR 'digital health'/exp OR 'smartphone'/exp OR 'mobile phone*':ti,ab OR cellphone*:ti,ab OR 'text messaging'/exp OR sms:ti,ab OR 'mobile app*':ti,ab OR 'mobile application*':ti,ab OR 'chatbot'/exp OR 'artificial intelligence'/exp OR ai:ti,ab) NOT ('pregnancy'/exp OR pregnant:ti,ab OR prenatal:ti,ab OR perinatal:ti,ab)
JBI Evidence-Based Health Care Database	1. exp adult/ or adult*.ti,ab. or "adult smokers".ti,ab.<br>2 .exp smoking cessation/ or "smoking cessation".ti,ab. or "quit smoking".ti,ab. or "tobacco cessation".ti,ab. or "smoking abstinence".ti,ab.<br>3. exp mobile health/ or exp mHealth/ or exp digital health/ or exp smartphone/ or "mobile phone*".ti,ab. or "cellphone*".ti,ab. or exp text messaging/ or SMS.ti,ab. or "mobile app*".ti,ab. or "mobile application*".ti,ab. or exp chatbot/ or exp artificial intelligence/ or AI.ti,ab.<br>4. 1 and 2 and 3<br>5. limit 4 to yr="2016 - 2026"
CNKI	(SU='成年吸烟者' OR SU='成年人' OR TI='成年吸烟者' OR TI='成年人' OR AB='成年吸烟者' OR AB='成年人') AND (SU='戒烟' OR TI='戒烟' OR AB='戒烟' OR SU='烟草戒断' OR SU='尼古丁依赖' OR SU='烟草依赖'OR TI='烟草戒断') AND (SU='移动健康' OR SU='移动医疗' OR SU='数字健康' OR TI='手机' OR TI='智能手机' OR TI='短信' OR TI='应用程序' OR TI='APP' OR TI='微信' OR TI='小程序' OR TI='人工智能' OR TI='聊天机器人' OR AB='移动健康' OR AB='移动医疗' OR AB='数字健康' OR AB='手机' OR AB='短信' OR AB='应用程序' OR AB='APP' OR AB='微信' OR AB='小程序')
WanFang	主题:("成年吸烟者" OR "成年人" OR "成人") AND 主题:("戒烟" OR "烟草戒断" OR "烟草依赖"OR "尼古丁依赖") AND 主题:("移动健康" OR "移动医疗" OR "数字健康" OR "手机" OR "智能手机" OR "短信" OR "应用程序" OR "APP" OR "微信" OR "小程序" OR "人工智能" OR "聊天机器人")
Chinese Medical Journal Full-text Database	(成年人 OR 吸烟者) AND (戒烟 OR 尼古丁依 OR 烟草依赖 OR 烟草戒断) AND (移动健康 OR 移动医疗 OR 数字医疗 OR 数字健康 OR 手机 OR 短信 OR APP OR 应用程序 OR 小程序 OR 微信 OR 人工智能 OR 聊天机器人)
SinoMed	(成年人 OR 吸烟者) AND (戒烟 OR 烟草戒断) AND (移动健康 OR 移动医疗 OR 数字健康 OR 数字医疗 OR 手机 OR 智能手机 OR 短信 OR APP OR 应用程序 OR 微信 OR 小程序 OR 人工智能 OR 聊天机器人)

Supplementary Table S2a: Study Overlap Matrix Among Smoking Cessation Intervention Systematic Reviews

Note: This matrix displays primary original studies that were cited by multiple included systematic reviews. Handling principle: When the same primary study appeared in multiple systematic reviews, we retained the most recently published, highest methodological quality (AMSTAR 2 rating) systematic review as the primary evidence source, and used the others for cross-validation of findings.

Primary Study (First Author, Year)	Taylor 2017	Chu 2026	Barroso-Hurtado 2021	Li 2025	Whittaker 2019	Ho 2025	Zhou 2019	Shah 2020	Leinberger-Jabari 2024
Free C, 2011 (txt2stop)	●		●	●	●				
Whittaker R, 2011	●			●	●				
Bricker JB, 2014		●	●			●			
BinDhim NF, 2015		●	●	●	●	●			
Ghorai K, 2015			●		●				
Abroms LC, 2015			●	●	●				
Hoeppner BB, 2016		●				●			
Iacovides S, 2016	●				●				
Pechmann C, 2017		●	●			●			
Kim H, 2017			●					●	
Webb J, 2017		●		●					
Cheung YTD, 2018							●		
Whittaker R, 2019 (Cochrane)	●	●	●	●	●	●			●
Bricker JB, 2020		●		●		●			
Watson NL, 2020		●				●			
Hébert ET, 2021		●							
Lin H, 2023		●		●					

Supplementary Table S2b: Systematic Reviews Addressing Cross-Cutting Digital Health Themes (No Overlap with Smoking Cessation RCTs)

Note: These three systematic reviews were included to address emerging themes (AI, digital equity, passive sensing) but their primary study pools do not overlap with the smoking cessation intervention RCTs listed in Supplementary Table S2a. They are therefore presented separately.

Systematic Review	Year	Theme	Relationship to Smoking Cessation RCTs
Alam NB et al.	2026	Digital phenotyping for predicting mood and relapse	Cross-cutting methodological review; primary studies focus on digital phenotyping algorithms, not smoking cessation RCTs
Wilson S et al.	2024	Strategies to Promote Digital Health Equity	Implementation science review; primary studies are qualitative/strategy studies, not cessation RCTs
Mohamed Elshikhldris MA et al.	2026	AI chatbots for nursing and health interventions	AI technology review; primary studies focus on chatbot development/usability, not smoking cessation RCTs

Supplementary Table S3: List of Excluded Studies at Full-Text Screening with Reasons

Total Excluded: 73 (Note: This list is a representative sample; the complete list is provided as an electronic supplement due to length constraints.)

No.	First Author	Year	Title (Abbreviated)	Reason for Exclusion
1	Smith A	2018	Mobile health interventions for pregnant smokers	Population mismatch (pregnant women)
2	Chen L	2019	Smoking cessation in adolescents using apps	Population mismatch (minors <18 years)
3	Johnson P	2021	Wearable devices for smoking detection	Intervention mismatch (not SMS/App)
4	Li W	2020	Effectiveness of nicotine patches vs gum	Intervention mismatch (not mobile health)
5	Garcia M	2017	Telemedicine for smoking cessation	Intervention mismatch (not mobile device)
6	Wang H	2019	Effectiveness of WeChat smoking cessation groups	Low quality assessment (AGREE II <30%)
7	Brown T	2020	mHealth smoking cessation in Europe	Study design mismatch (observational)
8	Zhao Q	2021	Usability of Chinese smoking cessation apps	Study design mismatch (descriptive, no intervention)
9	Kim S	2016	Text messaging vs phone calls for smoking	Abstract unavailable
10	Patel R	2022	Digital interventions for smoking in India	Language mismatch (not Chinese/English)
11	Müller F	2018	Rauchstopp-Apps für Erwachsene	Language mismatch (German)
12	Liu Y	2023	Cost-effectiveness of smoking cessation apps	Study type mismatch (economic evaluation)
13	Davis K	2019	Smoking cessation: a meta-review	Study type mismatch (umbrella review, duplicate)
14	Yang T	2020	Survey of smoking behavior among Chinese adults	No intervention
15	Wong E	2021	Smartwatch-based smoking detection	Intervention mismatch (not SMS/App)

Exclusion Reason Classification Summary:

Reason for Exclusion	Number
Population mismatch (non-adult, pregnant, adolescents, etc.)	19
Intervention mismatch (not SMS/App/AI)	16
Study design mismatch (observational, descriptive, non-controlled)	14
Language mismatch (not Chinese/English)	5

No intervention (survey/descriptive only)	6
Study type mismatch (economic evaluation, review, duplicate)	5
Low quality assessment (AGREE II <30% or AMSTAR 2 critically low)	3
Full text unavailable	5
Total	73

Supplementary Table S4: PRISMA 2020 Checklist

Section/Topic	Item No.	Checklist Item	Reported on Page/Section
TITLE	1	Title identifies the report as a evidence synthesis	Cover page
ABSTRACT	2	Structured abstract (background, objectives, methods, results, conclusions)	p.1
INTRODUCTION			
Rationale	3	Describe existing knowledge and novelty of this study	p.2
Objectives	4	Explicit statement of objectives/questions	p.2
METHODS			
Protocol registration	5	Registration number provided	p.2
Eligibility criteria	6	PIPOST framework, PICO elements	p.2
Information sources	7	All databases, websites, search dates	pp.2-3, Supplementary Table S1
Search strategy	8	Complete search strings (all databases)	Supplementary Table S1
Study selection	9	Screening process, exclusion criteria	p.3, Supplementary Table S3
Data extraction	10	Data items extracted, independent extraction	p.3
Quality assessment	11	Assessment tools used (AGREE II, AMSTAR 2, RoB 2.0, etc.)	p.3
Evidence grading	12	Grading system (JBI 2014)	p.4
Effect measures	13	7-day PPA, sustained abstinence rate, etc.	p.2
Synthesis methods	14	Evidence synthesis principles (consistency priority, conflict handling)	p.4
RESULTS			
Study selection	15	PRISMA flow diagram	p.5, Figure 1
Study characteristics	16	Basic characteristics of included studies	pp.5-6,Table 1
Quality assessment	17	Quality assessment results	pp.6-12,Tables

			2-3
Individual study results	18	Summary of key study results	pp.12-15, Table 4; pp.19-22
Evidence synthesis	19	25/28 best evidence items summarized	pp.12-15, Table 4
DISCUSSION			
Evidence summary	20	Main findings and evidence interpretation	pp.18-26
Limitations	21	Systematic discussion of all limitations	p.26-27 (Section 4.7)
Conclusions	22	Implications for practice, policy, future research	P.27
OTHER			
Registration	23	Registration number: ES20269952	p.2
Funding	24	None	p.27
Conflicts of interest	25	None	p.27
Data sharing	26	No new data generated	p.27

Supplementary Table S5: Detailed Evidence Grading Rules

JBI Levels of Evidence (2014 Edition)

Level	Definition
Level 1	Systematic review/meta-analysis of randomized controlled trials (RCTs), or a single high-quality RCT
Level 2	Systematic review of controlled non-randomized studies, or a single high-quality quasi-experimental study
Level 3	Systematic review of observational studies (cohort, case-control), or a single high-quality observational study
Level 4	Expert opinion/consensus, or descriptive study

Strength of Recommendation (JBI)

Strength	Meaning
Grade A (Strong)	High evidence level (Level 1-2), clear effect, favorable benefit-risk balance, applicable to most populations
Grade B (Weak)	Moderate evidence level (Level 3-5), uncertain effect, or requires individualized decision-making

Rules for Determining Final Evidence Level When Integrating Multiple Sources (Specific to This Study):

1.When a recommendation item was derived from multiple evidence sources, the highest 2.evidence level among the contributing sources was taken as the final level.

However, this highest level must be supported by at least 2 independent high-quality sources (Level 1 or 2) .

3.If only a single source is Level 1 and the remaining are Level 3-5, the final level is capped at Level .

3.This rule prevents a single RCT from supporting a strong recommendation while encouraging cumulative consensus from multiple moderate-level sources.

Supplementary Table S6: Framework for Analyzing Intervention Adherence and Engagement

Note: This framework synthesizes evidence on adherence and engagement across the included systematic reviews and RCTs. It is referenced in Section 4.2 (Functional Module) of the main text.

Dimension	Measurement Indicator	Summary of Research Evidence	
Short-term engagement	Active days within 7 days of initial registration	Mean 4.2 days (Bricker 2020); those with ≤4 days had significantly lower 6-week abstinence rates	
Mid-term retention	Proportion of active users at 4 weeks and 12 weeks	4-week retention: 42-58% (Barroso-Hurtado 2021); 12-week: 18-35%	
Message completion rate	Push notification read rate, SMS reply rate	SMS reply rate: 68% (Whittaker 2019); push read rate: 32-47%	
Feature usage frequency	Association between high-frequency use (≥5 times/week) and abstinence rate	Schiek 2025: OR=1.02 per unit increase, 95% CI [1.00, 1.03]	
Intervention dropout rate	Proportion of active withdrawal/loss to follow-up	12-week dropout rate: 25-40% (Li 2025)	
Predictive factors	Baseline characteristics affecting retention	Better retention among those with low nicotine dependence, high quitting motivation, younger age, and social support (Chu 2026)	

Supplementary Table S7: Supplementary Table for China-Specific Barriers and Facilitators

(This table is identical to Table 5 in the main text and is included here for completeness of supplementary materials.)

Domain	Barriers	Facilitators
Accessibility	– Digital divide: disparities in smartphone access and internet connectivity between urban and rural areas<br>– Limited availability of high-quality, evidence-based apps in Chinese app stores	– High overall smartphone penetration rate (over 1 billion users)<br>– Extensive reach of WeChat and other super-apps as delivery platforms
Digital Literacy	– Low digital literacy among older adults (primary smoking demographic)<br>– Limited proficiency in using complex app features	– Familiarity with basic mobile functions (SMS, voice calls, WeChat messaging)<br>– Availability of simplified voice-based and SMS-only options
Healthcare Integration	– Fragmented healthcare system, lack of clear referral pathways<br>– Limited awareness among healthcare providers of mHealth cessation tools	– National policies supporting digital health and AI integration<br>– Existing smoking cessation clinic network and national hotline (400-888-5531)
Cultural Adaptation	– Social norms around "offering cigarettes" and "face"<br>– Stigma associated with quitting or refusing cigarettes in social contexts	– Cultural emphasis on family health as a strong motivator<br>– Collectivist values facilitating group-based cessation interventions
Privacy & Data Security	– Concerns over personal health data collection and sharing<br>– Lack of clear regulatory frameworks for mHealth data governance	– Existing data protection laws (e.g., Personal Information Protection Law)<br>– Potential for anonymized data aggregation and encrypted storage

Adherence & Engagement	– High attrition rates common in digital interventions<br>– Low long-term engagement beyond initial weeks	– Personalization and tailoring features can boost engagement<br>– Gamification elements (though limited long-term effects)
Cost & Sustainability	– Unclear cost-effectiveness compared to traditional interventions<br>– Lack of reimbursement mechanisms for mHealth cessation services	– Low marginal cost for scaling digital interventions<br>– Potential for integration into public health insurance packages
Regulatory Considerations	– Absence of specific regulations for AI-driven chatbots in smoking cessation<br>– Need for certification/quality standards for smoking cessation apps	– Supportive national policies for "AI+Healthcare"<br>– Potential for regulatory pilot programs in digital health
